# No Influence of Dopamine System Gene Variations on Acute Effects of MDMA

**DOI:** 10.3389/fpsyt.2019.00755

**Published:** 2019-10-24

**Authors:** Patrick Vizeli, Matthias E. Liechti

**Affiliations:** Division of Clinical Pharmacology and Toxicology, Department of Biomedicine and Department of Clinical Research, University Hospital Basel, Basel, Switzerland

**Keywords:** dopamine, SCL6A3, DAT1, DRD2, DRD4, MDMA

## Abstract

3,4-Methylenedioxymethamphetamine (MDMA, ecstasy) is a recreational substance also investigated as medication for posttraumatic stress disorder. Dopamine (DA) system stimulation likely contributes to the acute mood effects of amphetamines, including MDMA. Genetic variants, such as single-nucleotide polymorphisms (SNPs), and polymorphic regions of the DA system genes may in part explain interindividual differences in the acute responses to MDMA in humans. We characterized the effects of common genetic variants within genes coding for key players in the DA system including the dopamine D2 receptor (DRD2/ANKK1 rs1800497, DRD2 rs6277, and rs107959), the dopamine transporter (DAT1 rs28363170, rs3836790, rs6347, rs11133767, rs11564774, rs460000, and rs463379), and dopamine D4 receptor [DRD4, variable-number tandem repeat (VNTR)] on the subjective and autonomic response to MDMA (125 mg) in pooled data from randomized, placebo-controlled, crossover studies in a total of 149 healthy subjects. Plasma concentrations of MDMA were used as covariate in the analysis to control for individual pharmacokinetic (metabolic and weight) differences. None of the tested genetic polymorphisms within the DA system altered effects of MDMA when adjusting for multiple comparisons. Genetic variations in genes coding for players of the DA system are unlikely to explain interindividual variations in the acute effects of MDMA in humans.

## Introduction

3,4-Methylenedioxymethamphetamine (MDMA; ecstasy) is widely used recreationally for its euphoric effects. Additionally, recent investigations are looking into MDMA as a medication to assist psychotherapy in patients with posttraumatic stress disorder (PTSD) (1–3). MDMA acts mainly as a releaser and reuptake inhibitor of serotonin (5-HT), norepinephrine (NE), and dopamine (DA) *via* an interaction with the respective transporter (4–7). The subjective effects of MDMA have been shown to mainly depend on transporter-mediated release of 5-HT and NE (8). In animals, however, the possibility was raised that the importance of interaction with the DA system would increase with the amount of drug taken (9). To what extent DA is mediating the acute effects of MDMA in humans is unclear. For example, the positive effects of MDMA were diminished after pharmacological inhibition of DA receptors with haloperidol (10). In addition, MDMA-induced hyperactivity was reduced in knockout mice without the DA receptor D_2_ gene (DRD2) (11). However, in contrast to the strongly diminished effect of MDMA in subjects with a blocked serotonin transporter, preventing the interaction of MDMA with the DA transporter (DAT) by pretreatment with bupropion or methylphenidate had no effect on the acute mood effects of MDMA in humans (12–15). Studies on the influence of genetic polymorphisms in the DA system could add adjuvant information to this matter and may also explore the role of the DA system in the interindividual differences in the response to MDMA. So far, only genetic variations of the enzymes that are involved in MDMA metabolism (mainly CYP2D6) displayed a robust influence on MDMA plasma levels in several clinical studies (16–18) and also showed a concomitant modulation of the pharmacodynamic effects of MDMA. However, genetic variants of pharmacological targets of MDMA may also alter its pharmacodynamic effects. A few studies explored the role of genetic polymorphisms of the 5-HT, NE, and oxytocin systems and found only minimal influences on acute effects of MDMA (19–23).

The DAT is a key target for many stimulant-type drugs, including cocaine, amphetamine, methylphenidate, and MDMA (6, 24). Additionally, the transporter is involved in various psychiatric disorders and treatment approaches (25–27). Subsequently, genetic polymorphisms within the single copy gene coding for the DAT (DAT1, SLC6A3) were investigated in relation to cocaine dependence and abuse, methamphetamine psychosis, attention-deficit/hyperactivity disorder (ADHD) and treatment, and bipolar disorder (28–36). Two common variable-number tandem repeat (VNTR) polymorphisms were most extensively studied. One, the rs28363170, is located in the 3′ untranslated region (3′UTR) of the DAT1 gene and exhibits 9 or 10 repeats as most common forms (37). Homozygous carriers of the 9-repeat allele were found to be at a higher risk for persistent ADHD, and the 10/10 genotype was associated with ADHD in children (38). Subjects with the 9/9 genotype were less susceptible to the subjective effects of amphetamine (39). However, carriers of at least one 9-repeat allele showed higher ratings of “high,” “any drug effect,” “anxious,” and “stimulated” after cocaine (40). Conversely, homozygous 10-repeat carriers in combination with a 5-repeat allele of the other extensively studied VNTR in the DAT1, the rs3836790, displayed a lower response to “good drug effects,” “bad drug effects,” “depressed,” and “anxious” (40). The rs3836790 VNTR is located in intron 8 of the human DAT1 gene. The most common forms of this VNTR are 5 or 6 repeats (30). A study in a Brazilian sample found a positive association of the 6-repeat allele and cocaine addiction (28). In contrast, another yet smaller case-control study in a Spain sample showed an overrepresentation of the 5/5 genotype in cocaine abusers (33).

MDMA also directly and indirectly interacts with DA receptors (4). Especially the inhibition of the D_2_ with haloperidol showed a significant reduction in MDMA positive effects (10). MDMA-unrelated pharmacogenetic studies showed a positive association of the minor allele of the DRD2 single-nucleotide polymorphisms (SNPs) rs1079597 and rs1800497 with heroin dependence (41), rs6277 and rs1800497 with nicotine dependence (42), and rs6277 with alcohol dependence in males (43). The VNTR polymorphism within the gene coding for the subtype 4 of the DA receptors (DRD4) is also frequently studied in relation to psychiatric disorders and personality traits (44–47). DRD4 VNTR variations range from 2 repeats to 10 repeats, with 4 and 7 repeats as the most frequent forms (48). The presence of a 7-repeat allele has been linked with personal traits like high novelty seeking, risky decision making, and broad sexual interest (44, 47). Moreover, children and adolescents suffering from ADHD and carrying the 7-repeat allele had to take higher doses of methylphenidate to reach sufficient efficacy (49). This finding is in line with earlier results from an *in vitro* study showing a reduced sensitivity of the 7-repeat allele toward DA compared with the 2- and 4-repeat allele (50).

The present study is the first to explore the influence of variants within genes coding for the DA system on the acute effects of MDMA in humans. We analyzed DRD2/ANKK1 rs1800497, DRD2 rs6277, and rs107959, DAT1 rs28363170, rs3836790, rs6347, rs11133767, rs11564774, rs460000, and rs463379, DRD4 VNTR and their influence on acute subjective and autonomic effects of MDMA. Given the partially inconclusive pharmacogenetic studies in addition to the unclear degree to which MDMA effects are driven by the interaction with the DA system, we hypothesized that genetic mutations of the DA system would not influence cardiostimulant effects and have only minimal influence on the mood effects of MDMA.

## Methods

### Study Design

This was a pooled analysis of nine double-blind, placebo-controlled, crossover studies that used similar methods and were conducted in healthy subjects and in the same laboratory (14, 15, 51–55). The studies included a total of 164 healthy subjects. Seven studies included 16 subjects each, for a total of 112 subjects, who received 125 mg MDMA twice, once alone and once after pretreatment with a medication (14, 15, 51–54). Two additional studies included 24 and 28 subjects who received 125 mg MDMA alone, placebo, or other treatments (55; Holze et al., unpublished). In the present analysis, only data from the MDMA-alone and placebo sessions were used. In all of the studies, the washout periods between single-dose administrations of MDMA were at least 7 days to exclude possible carryover effects. The studies were all registered at ClinicalTrials.gov (NCT00886886, NCT00990067, NCT01136278, NCT01270672, NCT01386177, NCT01465685, NCT01771874, NCT01951508, and NCT03019822). All of the studies were approved by the local ethics committee and, if necessary, Swiss Agency for Therapeutic Products (Swissmedic). The studies were conducted in accordance with the Declaration of Helsinki. MDMA administration in healthy subjects was authorized by the Swiss Federal Office for Public Health (BAG), Bern, Switzerland. Written informed consent was obtained from all of the participants. All of the subjects were paid for their participation. Detailed pharmacokinetic and safety data from these studies have been reported elsewhere (17, 18, 56). Test sessions were conducted in a quiet hospital research ward with no more than two research subjects present per session. The participants were comfortably lying in hospital beds and were mostly listening to music and not engaging in physical activities. MDMA was given without food in the fasting state in the morning at 8:00–9:00 AM. A small standardized lunch was served at 12:00–1:00 PM.

### Subjects

A total of 164 healthy subjects of European descent, 18–45 years old (mean ± SD = 25.3 ± 4 years), were recruited from the University of Basel campus and participated in the study. One genotyping sample was missing, three participants did not give consent for genotyping, and 11 subjects participated twice (only one participation that included all outcome measures was used), resulting in a final data set of 149 subjects (76 women). The mean ± SD body weight was 69 ± 11 kg (range: 46–97 kg).

The exclusion criteria included a history of psychiatric disorders, physical illness, a lifetime history of illicit drug use more than 10 times (with the exception of past cannabis use), illicit drug use within the past 2 months, and illicit drug use during the study, as determined by urine tests that were conducted before the test sessions, as reported in detail elsewhere (52–54). Fifty-five subjects had prior illicit drug experiences (1–8 times), of which 27 subjects had previously used MDMA (1–5 times), 14 subjects had previously used amphetamine or methamphetamine (1–2 times), 11 subjects had previously used cocaine (1–4 times), eight subjects had previously used lysergic acid diethylamide (1–2 times), and 11 subjects had previously used psilocybin (1–4 times).

### Study Drug

(±)MDMA hydrochloride (Lipomed AG, Arlesheim, Switzerland) was administered orally in a single dose of 125 mg prepared as gelatin capsules (25 and 100 mg). Similar amounts of MDMA are found in ecstasy pills (57) and have been used in clinical trials in patients (1, 2). The doses were not adjusted for body weight or sex. The dose per body weight (mean ± SD) was 1.9 ± 0.3 mg/kg (range: 1.3–2.7 mg/kg).

### Physiological Effects

Blood pressure, heart rate, and body temperature were assessed repeatedly before and 0, 0.33, 0.67, 1, 1.5, 2, 2.5, 3, 4, 5, and 6 h after MDMA or placebo administration. Systolic and diastolic blood pressures and heart rate were measured using an automatic oscillometric device (OMRON Healthcare Europe NA, Hoofddorp, Netherlands). The measurements were performed in duplicate at an interval of 1 min and after a resting time of at least 10 min. The averages were calculated for the analysis. Mean arterial pressure (MAP) was calculated as diastolic blood pressure + (systolic blood pressure – diastolic blood pressure)/3. The rate pressure product (RPP) was calculated as systolic blood pressure × heart rate. Core (tympanic) temperature was measured using a Genius 2 ear thermometer (Tyco Healthcare Group LP, Watertown, NY, USA). In two studies (N = 46), the 2-h time point was not used.

### Pharmacodynamic Measures

Visual Analog Scales (VASs) were repeatedly used to assess subjective effects over time (58). The VASs included for instance “any drug effect,” “good drug effect,” and “stimulated.” The VASs were presented as 100 mm horizontal lines (0–100%), marked from “not at all” on the left to “extremely” on the right. Subjective effects like “concentration,” “appetite,” “tired,” “want to be hugged,” “want to hug,” and “talkative” were bidirectional (±50 mm). Not all VAS components were presented in all studies. Exact numbers of subjects per genotype group are reported in **Tables 1**–**3**. The VASs were applied before and 0, 0.33, 0.67, 1, 1.5, 2, 2.5, 3, 4, 5, and 6 h after MDMA or placebo administration. In two studies (N = 46), the 2-h time point is missing; additionally, in one study (N = 21), the 2.5-h time point is also missing.

**Table 1:** Effects of polymorphisms in the dopamine receptor D2 gene on the maximal response to 125 mg MDMA (mean ± SD (N) and statistics) corrected with MDMA AUC_6_ (exclusive plasma concentrations).

DRD2/ANKK1 rs1800497	AA	AG	GG	F	p value	p value^a^	η^2^
N	2	46	101				
Female, N [%]	1 (50)	30 [65]	45 [45]				
Drug experience, N [%]	2 (100)	17 [37]	36 [36]				
MDMA plasma concentration Cmax, ng/ml	236 ± 76 (2)	239 ± 47 (46)	223 ± 49 (101)	1.71	NS	NS	0.023
MDMA plasma concentration AUC_6_, ng*h/ml	964 ± 235 (2)	994 ± 199 (46)	944 ± 205 (101)	0.97	NS	NS	0.013
Visual Analog Scale rating ΔE_max_							
Any drug effect	84 ± 18 (2)	77 ± 23 (46)	70 ± 28 (101)	0.74	NS	NS	0.008
Good drug effect	94 ± 9 (2)	78 ± 26 (46)	70 ± 30 (101)	1.31	NS	NS	0.016
Bad drug effect	24 ± 35 (2)	21 ± 25 (46)	14 ± 24 (101)	1.14	NS	NS	0.015
Drug liking	96 ± 6 (2)	78 ± 28 (46)	72 ± 29 (101)	1.03	NS	NS	0.013
Stimulated	91 ± 13 (2)	68 ± 32 (46)	59 ± 35 (101)	1.44	NS	NS	0.018
High mood	96 ± 6 (2)	73 ± 30 (46)	66 ± 34 (101)	0.98	NS	NS	0.012
Concentration	28 ± 31 (2)	6.2 ± 14 (46)	9.2 ± 16 (101)	2.09	NS	NS	0.028
Talkative	48 ± 4 (2)	18 ± 20 (46)	22 ± 18 (101)	3.12	0.047*	NS	0.040
Appetite	7.5 ± 9.2 (2)	-5.3 ± 39 (23)	-8.9 ± 27 (47)	0.43	NS	NS	0.012
Tired	24 ± 8 (2)	19 ± 34 (33)	20 ± 32 (74)	0.07	NS	NS	0.001
Fear	7.0 ± 9.9 (2)	7.3 ± 15 (31)	5.9 ± 17 (64)	0.06	NS	NS	0.001
Happy	50 (1)	26 ± 19 (32)	27 ± 19 (73)	0.63	NS	NS	0.011
Want to be hugged	NA (0)	13 ± 18 (23)	13 ± 19 (54)	0.12	NS	NS	0.001
Want to hug	NA (0)	14 ± 17 (23)	13 ± 18 (54)	0.02	NS	NS	0.000
Vital signs parameters ΔE_max_							
Systolic blood pressure, mmHg	21 ± 31 (2)	25 ± 11 (46)	23 ± 13 (101)	0.32	NS	NS	0.004
Diastolic blood pressure, mmHg	13 ± 11 (2)	15 ± 10 (46)	13 ± 9 (101)	0.78	NS	NS	0.010
Mean arterial pressure, mmHg	14 ± 22 (2)	19 ± 10 (46)	16 ± 9 (101)	0.91	NS	NS	0.011
Heat rate beat/min	31 ± 33 (2)	20 ± 15 (46)	16 ± 13 (101)	2.07	NS	NS	0.027
Rate pressure product, mmHg/min	6,343 ± 6,658 (2)	4,967 ± 2,855 (46)	4,203 ± 2,776 (101)	1.26	NS	NS	0.017
Body temperature, °C	0.5 ± 0.1 (2)	0.2 ± 0.4 (46)	0.2 ± 0.5 (101)	0.34	NS	NS	0.005
Adjective Mood Rating Scale rating ΔE_max_							
Activity	10 ± 8 (2)	2.1 ± 4.2 (46)	2.4 ± 5.3 (101)	2.40	0.09	NS	0.032
High mood	7.5 ± 0.7 (2)	2.2 ± 2.8 (46)	3.0 ± 3.2 (101)	3.49	0.033*	NS	0.046
Fear/depression	-1.5 ± 2.1 (2)	1.2 ± 3.3 (46)	1.2 ± 3.4 (101)	0.63	NS	NS	0.009
DRD2 rs6277	AA	AG	GG	F	p value	p value^a^	η^2^
N	50	73	26				
Female, N [%]	25 [50]	39 [53]	12 [46]				
Drug experience, N [%]	18 [36]	29 [40]	8 [31]				
MDMA plasma concentration Cmax, ng/ml	225 ± 52 (50)	233 ± 47 (73)	221 ± 47 (26)	0.70	NS	NS	0.009
MDMA plasma concentration AUC_6_, ng*h/ml	949 ± 213 (50)	974 ± 203 (73)	939 ± 186 (26)	0.38	NS	NS	0.005
Visual Analog Scale rating ΔE_max_							
Any drug effect	70 ± 31 (50)	73 ± 25 (73)	77 ± 21 (26)	0.95	NS	NS	0.011
Good drug effect	72 ± 31 (50)	74 ± 26 (73)	71 ± 31 (26)	0.04	NS	NS	0.000
Bad drug effect	11 ± 21 (50)	16 ± 24 (73)	25 ± 30 (26)	3.42	0.036*	NS	0.043
Drug liking	74 ± 30 (50)	74 ± 26 (73)	73 ± 34 (26)	0.01	NS	NS	0.000
Stimulated	57 ± 37 (50)	63 ± 33 (73)	71 ± 32 (26)	1.63	NS	NS	0.020
High mood	67 ± 33 (50)	70 ± 32 (73)	67 ± 36 (26)	0.09	NS	NS	0.001
Concentration	10 ± 16 (50)	6.3 ± 15 (73)	12 ± 18 (26)	1.59	NS	NS	0.021
Talkative	22 ± 18 (50)	20 ± 19 (73)	22 ± 20 (26)	0.25	NS	NS	0.003
Appetite	-17 ± 32 (26)	-0.8 ± 30 (35)	-4.5 ± 25 (11)	2.26	NS	NS	0.061
Tired	23 ± 33 (36)	20 ± 33 (55)	14 ± 29 (18)	0.44	NS	NS	0.008
Fear	4.5 ± 10 (35)	5.7 ± 14 (47)	13 ± 29 (15)	1.64	NS	NS	0.034
Happy	29 ± 18 (32)	26 ± 20 (53)	27 ± 18 (21)	0.30	NS	NS	0.005
Want to be hugged	13 ± 18 (24)	13 ± 19 (38)	12 ± 17 (15)	0.08	NS	NS	0.002
Want to hug	12 ± 17 (24)	14 ± 19 (38)	13 ± 16 (15)	0.01	NS	NS	0.000
Vital signs parameters ΔE_max_							
Systolic blood pressure, mmHg	24 ± 13 (50)	23 ± 13 (73)	24 ± 11 (26)	0.10	NS	NS	0.001
Diastolic blood pressure, mmHg	13 ± 9 (50)	14 ± 8 (73)	14 ± 13 (26)	0.08	NS	NS	0.001
Mean arterial pressure, mmHg	17 ± 10 (50)	17 ± 9 (73)	18 ± 11 (26)	0.16	NS	NS	0.002
Heat rate beat/min	19 ± 14 (50)	16 ± 15 (73)	20 ± 14 (26)	1.57	NS	NS	0.021
Rate pressure product, mmHg/min	4,635 ± 2,630 (50)	4,211 ± 3,111 (73)	4,867 ± 2,541 (26)	0.85	NS	NS	0.011
Body temperature, °C	0.3 ± 0.6 (50)	0.2 ± 0.5 (73)	0.2 ± 0.4 (26)	0.42	NS	NS	0.006
Adjective Mood Rating Scale rating ΔE_max_							
Activity	2.6 ± 5.7 (50)	2.2 ± 4.9 (73)	2.7 ± 4.5 (26)	0.15	NS	NS	0.002
High mood	3.1 ± 3.2 (50)	2.9 ± 3.2 (73)	2.1 ± 3.0 (26)	0.84	NS	NS	0.012
Fear/depression	0.7 ± 3 (50)	1.3 ± 3.1 (73)	1.5 ± 4.5 (26)	0.79	NS	NS	0.011
DRD2 rs1079597	CC	CT	TT	F	p value	p value^a^	η^2^
N	111	37	1				
Female, N [%]	53 [48]	22 [59]	1 [100]				
Drug experience, N [%]	40 [36]	14 [38]	1 [100]				
MDMA plasma concentration Cmax, ng/ml	226 ± 49 (111)	234 ± 49 (37)	290 (1)	1.19	NS	NS	0.016
MDMA plasma concentration AUC_6_, ng*h/ml	949 ± 202 (111)	985 ± 208 (37)	1130 (1)	0.78	NS	NS	0.011
Visual Analog Scale rating ΔE_max_							
Any drug effect	71 ± 28 (111)	76 ± 21 (37)	96 (1)	0.44	NS	NS	0.005
Good drug effect	71 ± 30 (111)	76 ± 25 (37)	100 (1)	0.44	NS	NS	0.006
Bad drug effect	13 ± 24 (111)	25 ± 26 (37)	0 (1)	3.09	0.049*	NS	0.039
Drug liking	73 ± 29 (111)	77 ± 28 (37)	100 (1)	0.40	NS	NS	0.005
Stimulated	59 ± 35 (111)	71 ± 31 (37)	100 (1)	1.64	NS	NS	0.020
High mood	68 ± 33 (111)	70 ± 32 (37)	100 (1)	0.28	NS	NS	0.004
Concentration	8.9 ± 16 (111)	6.3 ± 15 (37)	50 (1)	3.92	0.022*	NS	0.051
Talkative	22 ± 18 (111)	19 ± 21 (37)	50 (1)	1.58	NS	NS	0.020
Appetite	-11 ± 29 (53)	2.4 ± 35 (18)	1.0 (1)	1.35	NS	NS	0.037
Tired	21 ± 32 (80)	18 ± 34 (28)	30 (1)	0.14	NS	NS	0.002
Fear	5.5 ± 16 (73)	9.5 ± 17 (23)	0 (1)	0.61	NS	NS	0.013
Happy	27 ± 19 (78)	26 ± 19 (27)	50 (1)	0.62	NS	NS	0.011
Want to be hugged	13 ± 18 (58)	14 ± 19 (19)	NA (0)	0.01	NS	NS	0.000
Want to hug	13 ± 18 (58)	15 ± 18 (19)	NA (0)	0.03	NS	NS	0.000
Vital signs parameters ΔE_max_							
Systolic blood pressure, mmHg	23 ± 13 (111)	26 ± 11 (37)	43 (1)	1.52	NS	NS	0.019
Diastolic blood pressure, mmHg	13 ± 9 (111)	15 ± 11 (37)	20 (1)	0.65	NS	NS	0.008
Mean arterial pressure, mmHg	16 ± 9 (111)	19 ± 10 (37)	29 (1)	1.33	NS	NS	0.017
Heat rate beat/min	16 ± 14 (111)	20 ± 14 (37)	54 (1)	3.89	0.023*	NS	0.050
Rate pressure product, mmHg/min	4,240 ± 2,838 (111)	4,972 ± 2,700 (37)	11,050 (1)	3.24	0.042*	NS	0.041
Body temperature, °C	0.2 ± 0.5 (111)	0.3 ± 0.4 (37)	0.6 (1)	0.65	NS	NS	0.009
Adjective Mood Rating Scale rating ΔE_max_							
Activity	2.4 ± 5.3 (111)	2.0 ± 3.9 (37)	16 (1)	3.74	0.026*	NS	0.049
High mood	3 ± 3.2 (111)	2.3 ± 2.8 (37)	8.0 (1)	1.92	NS	NS	0.026
Fear/depression	1.1 ± 3.2 (111)	1.2 ± 3.7 (37)	0 (1)	0.07	NS	NS	0.001

N, number of subjects; SD, standard deviation; NS, not significant; Δ, values are change scores from placebo; ^a^p value additionally corrected for multiple comparisons according to the Nyholt method; η^2^, eta square; *, uncorrected p < 0.05.

**Table 2:** Effects of polymorphisms in the dopamine transporter 1 gene on the maximal response to 125 mg MDMA (mean ± SD (N) and statistics) corrected with MDMA AUC_6_ (exclusive plasma concentrations).

DAT1 3’-UTR rs28363170	99	910	1010	F	p value	p value^a^	η^2^
N	8	56	79				
Female, N [%]	2 [25]	29 [52]	41 [52]				
Drug experience, N [%]	4 [50]	18 [32]	31 [39]				
MDMA plasma concentration Cmax, ng/ml	221 ± 44 (8)	227 ± 46 (56)	230 ± 50 (79)	0.14	NS	NS	0.002
MDMA plasma concentration AUC_6_, ng*h/ml	939 ± 183 (8)	958 ± 180 (56)	961 ± 214 (79)	0.04	NS	NS	0.001
Visual Analog Scale rating ΔE_max_							
Any drug effect	78 ± 20 (8)	73 ± 26 (56)	71 ± 28 (79)	0.48	NS	NS	0.006
Good drug effect	84 ± 21 (8)	73 ± 28 (56)	71 ± 30 (79)	0.97	NS	NS	0.013
Bad drug effect	11 ± 21 (8)	15 ± 29 (56)	16 ± 22 (79)	0.14	NS	NS	0.002
Drug liking	83 ± 22 (8)	77 ± 26 (56)	70 ± 31 (79)	1.26	NS	NS	0.017
Stimulated	63 ± 35 (8)	60 ± 35 (56)	63 ± 35 (79)	0.09	NS	NS	0.001
High mood	79 ± 32 (8)	68 ± 34 (56)	67 ± 33 (79)	0.57	NS	NS	0.008
Concentration	14 ± 22 (8)	7.3 ± 16 (56)	9.3 ± 16 (79)	0.76	NS	NS	0.011
Talkative	23 ± 16 (8)	19 ± 18 (56)	22 ± 19 (79)	0.56	NS	NS	0.008
Appetite	-20 ± 25 (7)	-2.6 ± 37 (28)	-7 ± 26 (35)	1.14	NS	NS	0.032
Tired	6.9 ± 37 (7)	17 ± 31 (44)	25 ± 33 (53)	1.38	NS	NS	0.026
Fear	6.3 ± 21 (7)	4.1 ± 9.5 (33)	8 ± 19 (55)	0.59	NS	NS	0.013
Happy	15 ± 16 (3)	27 ± 20 (40)	27 ± 18 (59)	0.37	NS	NS	0.007
Want to be hugged	0 (1)	16 ± 20 (28)	10 ± 16 (44)	1.16	NS	NS	0.030
Want to hug	0 (1)	16 ± 19 (28)	11 ± 16 (44)	1.01	NS	NS	0.027
Vital signs parameters ΔE_max_							
Systolic blood pressure, mmHg	27 ± 15 (8)	25 ± 11 (56)	22 ± 14 (79)	1.06	NS	NS	0.014
Diastolic blood pressure, mmHg	21 ± 18 (8)	14 ± 8 (56)	12 ± 9 (79)	3.84	0.024*	NS	0.049
Mean arterial pressure, mmHg	25 ± 14 (8)	18 ± 8 (56)	16 ± 10 (79)	3.79	0.025*	NS	0.048
Heat rate beat/min	16 ± 8 (8)	18 ± 15 (56)	16 ± 15 (79)	0.28	NS	NS	0.004
Rate pressure product, mmHg/min	4,623 ± 2,214 (8)	4,684 ± 2,835 (56)	4,194 ± 2,970 (79)	0.54	NS	NS	0.007
Body temperature, °C	0 ± 0.3 (8)	0.3 ± 0.5 (56)	0.2 ± 0.5 (79)	1.34	NS	NS	0.019
Adjective Mood Rating Scale rating ΔE_max_							
Activity	4.0 ± 5.2 (8)	2.0 ± 4.9 (56)	2.3 ± 5.2 (79)	0.53	NS	NS	0.008
High mood	2.6 ± 1.5 (8)	3.0 ± 3.2 (56)	2.7 ± 3.3 (79)	0.16	NS	NS	0.002
Fear/depression	0.5 ± 2 (8)	1.5 ± 3.6 (56)	1 ± 3 (79)	0.64	NS	NS	0.009
**DAT1 Intron 8 rs3836790**	**55**	**56**	**66**	**F**	**p value**	**p value** **^a^**	**η** **^2^**
DAT1 rs6347	CC	CT	TT	F	p value	p value^a^	η^2^
N	7	54	85				
Female, N [%]	3 [43]	25 [46]	46 [54]				
Drug experience, N [%]	4 [57]	21 [39]	29 [34]				
MDMA plasma concentration Cmax, ng/ml	218 ± 43 (7)	225 ± 54 (54)	231 ± 45 (85)	0.48	NS	NS	0.007
MDMA plasma concentration AUC_6_, ng*h/ml	894 ± 201 (7)	945 ± 211 (54)	972 ± 196 (85)	0.65	NS	NS	0.009
Visual Analog Scale rating ΔE_max_							
Any drug effect	66 ± 20 (7)	69 ± 29 (54)	75 ± 26 (85)	0.59	NS	NS	0.007
Good drug effect	69 ± 26 (7)	72 ± 30 (54)	74 ± 28 (85)	0.05	NS	NS	0.001
Bad drug effect	-0.7 ± 19 (7)	18 ± 29 (54)	16 ± 21 (85)	1.57	NS	NS	0.020
Drug liking	79 ± 20 (7)	73 ± 29 (54)	74 ± 30 (85)	0.24	NS	NS	0.003
Stimulated	58 ± 33 (7)	58 ± 35 (54)	66 ± 34 (85)	0.70	NS	NS	0.009
High mood	65 ± 27 (7)	66 ± 36 (54)	71 ± 31 (85)	0.19	NS	NS	0.003
Concentration	-0.6 ± 5 (7)	9.2 ± 18 (54)	8.5 ± 15 (85)	1.18	NS	NS	0.016
Talkative	15 ± 15 (7)	20 ± 19 (54)	22 ± 19 (85)	0.37	NS	NS	0.005
Appetite	-21 ± 29 (3)	-9.8 ± 34 (25)	-4.9 ± 30 (43)	0.49	NS	NS	0.014
Tired	-10 ± 28 (5)	20 ± 33 (41)	22 ± 31 (61)	1.82	NS	NS	0.032
Fear	1.7 ± 2.9 (3)	4.7 ± 14 (35)	7.7 ± 18 (58)	0.48	NS	NS	0.010
Happy	20 ± 16 (4)	26 ± 19 (42)	29 ± 19 (57)	0.28	NS	NS	0.005
Want to be hugged	9.8 ± 20 (4)	13 ± 19 (29)	14 ± 18 (42)	0.05	NS	NS	0.001
Want to hug	8.5 ± 17 (4)	14 ± 19 (29)	14 ± 18 (42)	0.06	NS	NS	0.001
Vital signs parameters ΔE_max_							
Systolic blood pressure, mmHg	31 ± 7 (7)	24 ± 12 (54)	23 ± 14 (85)	2.00	NS	NS	0.026
Diastolic blood pressure, mmHg	18 ± 6 (7)	15 ± 11 (54)	12 ± 8 (85)	2.99	0.05	NS	0.038
Mean arterial pressure, mmHg	23 ± 6 (7)	18 ± 10 (54)	16 ± 10 (85)	3.67	0.028*	NS	0.046
Heat rate beat/min	16 ± 5 (7)	19 ± 15 (54)	17 ± 14 (85)	0.38	NS	NS	0.005
Rate pressure product, mmHg/min	4,394 ± 1,421 (7)	4,878 ± 2,775 (54)	4,264 ± 2,999 (85)	0.96	NS	NS	0.013
Body temperature, °C	0.6 ± 0.6 (7)	0.3 ± 0.5 (54)	0.2 ± 0.5 (85)	2.59	0.08	NS	0.035
Adjective Mood Rating Scale rating ΔE_max_							
Activity	1.7 ± 3.1 (7)	2.2 ± 5.9 (54)	2.4 ± 4.7 (85)	0.08	NS	NS	0.001
High mood	2.3 ± 1.9 (7)	2.7 ± 3.3 (54)	3.0 ± 3.2 (85)	0.29	NS	NS	0.004
Hear/depression	-1.1 ± 2.8 (7)	1.4 ± 3.2 (54)	1.2 ± 3.5 (85)	1.80	NS	NS	0.025
DAT1 rs11133767	CC	CT	TT	F	p value	p value^a^	η^2^
N	12	60	77				
Female, N [%]	6 [50]	29 [48]	41 [53]				
Drug experience, N [%]	5 [42]	23 [38]	27 [35]				
MDMA plasma concentration Cmax, ng/ml	225 ± 46 (12)	224 ± 50 (60)	232 ± 48 (77)	0.45	NS	NS	0.006
MDMA plasma concentration AUC_6_, ng*h/ml	933 ± 221 (12)	947 ± 192 (60)	973 ± 210 (77)	0.39	NS	NS	0.005
Visual Analog Scale rating ΔE_max_							
Any drug effect	68 ± 24 (12)	70 ± 29 (60)	76 ± 25 (77)	0.73	NS	NS	0.008
Good drug effect	70 ± 26 (12)	71 ± 30 (60)	74 ± 28 (77)	0.12	NS	NS	0.002
Bad drug effect	3.9 ± 18 (12)	17 ± 29 (60)	17 ± 21 (77)	1.47	NS	NS	0.019
Drug liking	77 ± 23 (12)	72 ± 30 (60)	75 ± 29 (77)	0.16	NS	NS	0.002
Stimulated	65 ± 29 (12)	56 ± 36 (60)	67 ± 33 (77)	1.37	NS	NS	0.017
High mood	68 ± 28 (12)	66 ± 36 (60)	71 ± 31 (77)	0.16	NS	NS	0.002
Concentration	3.7 ± 10 (12)	8.4 ± 18 (60)	9.4 ± 15 (77)	0.69	NS	NS	0.009
Talkative	22 ± 15 (12)	19 ± 20 (60)	22 ± 18 (77)	0.29	NS	NS	0.004
Appetite	-4.1 ± 25 (7)	-13 ± 33 (27)	-3.8 ± 30 (38)	0.71	NS	NS	0.020
Tired	12 ± 36 (10)	20 ± 31 (42)	22 ± 33 (57)	0.28	NS	NS	0.005
Fear	1.0 ± 1.9 (7)	4.6 ± 13 (40)	8.5 ± 19 (50)	1.09	NS	NS	0.023
Happy	25 ± 15 (7)	25 ± 20 (45)	29 ± 19 (54)	0.36	NS	NS	0.007
Want to be hugged	11 ± 17 (5)	11 ± 18 (33)	15 ± 19 (39)	0.30	NS	NS	0.008
Want to hug	10 ± 15 (5)	10 ± 17 (33)	16 ± 18 (39)	0.70	NS	NS	0.017
Vital signs parameters ΔE_max_							
Systolic blood pressure, mmHg	29 ± 8 (12)	24 ± 13 (60)	23 ± 13 (77)	1.87	NS	NS	0.024
Diastolic blood pressure, mmHg	17 ± 7 (12)	14 ± 11 (60)	12 ± 8 (77)	2.19	NS	NS	0.027
Mean arterial pressure, mmHg	22 ± 6 (12)	18 ± 10 (60)	16 ± 10 (77)	2.87	0.06	NS	0.035
Heat rate beat/min	16 ± 12 (12)	17 ± 15 (60)	18 ± 14 (77)	0.03	NS	NS	0.000
Rate pressure product, mmHg/min	4,592 ± 2,500 (12)	4,550 ± 2,840 (60)	4,384 ± 2,950 (77)	0.14	NS	NS	0.002
Body temperature, °C	0.4 ± 0.6 (12)	0.2 ± 0.5 (60)	0.2 ± 0.5 (77)	1.13	NS	NS	0.015
Adjective Mood Rating Scale rating ΔE_max_							
Activity	3.3 ± 5.1 (12)	2.3 ± 5.7 (60)	2.4 ± 4.6 (77)	0.23	NS	NS	0.003
High mood	3.7 ± 2.6 (12)	2.6 ± 3.2 (60)	2.9 ± 3.2 (77)	0.66	NS	NS	0.009
Fear/depression	-0.8 ± 2 (12)	1.2 ± 3.3 (60)	1.4 ± 3.4 (77)	2.28	NS	NS	0.030
DAT1 rs11564774	CC	CG	GG	F	p value	p value^a^	η^2^
N	62	66	20				
Female, N [%]	33 [53]	35 [53]	7 [35]				
Drug experience, N [%]	22 [35]	18 [27]	14 [70]				
MDMA plasma concentration Cmax, ng/ml	230 ± 48 (62)	230 ± 48 (66)	213 ± 50 (20)	1.05	NS	NS	0.014
MDMA plasma concentration AUC_6_, ng*h/ml	965 ± 210 (62)	977 ± 200 (66)	876 ± 179 (20)	1.98	NS	NS	0.027
Visual Analog Scale rating ΔE_max_							
Any drug effect	75 ± 25 (62)	69 ± 28 (66)	74 ± 23 (20)	1.70	NS	NS	0.019
Good drug effect	74 ± 28 (62)	70 ± 30 (66)	77 ± 26 (20)	1.38	NS	NS	0.018
Bad drug effect	18 ± 23 (62)	14 ± 25 (66)	16 ± 29 (20)	0.73	NS	NS	0.009
Drug liking	74 ± 31 (62)	72 ± 29 (66)	82 ± 23 (20)	1.72	NS	NS	0.022
Stimulated	68 ± 32 (62)	57 ± 36 (66)	63 ± 35 (20)	2.37	0.10	NS	0.029
High mood	71 ± 32 (62)	65 ± 34 (66)	72 ± 34 (20)	1.40	NS	NS	0.018
Concentration	9.5 ± 16 (62)	7.8 ± 16 (66)	8.6 ± 16 (20)	0.19	NS	NS	0.003
Talkative	23 ± 19 (62)	19 ± 19 (66)	21 ± 17 (20)	0.61	NS	NS	0.008
Appetite	-2.9 ± 29 (32)	-8.6 ± 30 (27)	-15 ± 35 (13)	1.21	NS	NS	0.034
Tired	25 ± 34 (45)	19 ± 28 (47)	10 ± 37 (17)	1.13	NS	NS	0.020
Fear	8.9 ± 20 (44)	1.8 ± 4.9 (37)	11 ± 20 (15)	2.57	0.08	NS	0.053
Happy	27 ± 19 (42)	26 ± 19 (52)	30 ± 17 (11)	0.87	NS	NS	0.016
Want to be hugged	10 ± 16 (30)	15 ± 19 (39)	14 ± 22 (7)	0.44	NS	NS	0.011
Want to hug	11 ± 16 (30)	15 ± 18 (39)	14 ± 22 (7)	0.38	NS	NS	0.010
Vital signs parameters ΔE_max_							
Systolic blood pressure, mmHg	22 ± 13 (62)	25 ± 13 (66)	23 ± 12 (20)	1.43	NS	NS	0.018
Diastolic blood pressure, mmHg	12 ± 8 (62)	14 ± 9 (66)	15 ± 12 (20)	1.83	NS	NS	0.023
Mean arterial pressure, mmHg	16 ± 9 (62)	18 ± 9 (66)	19 ± 11 (20)	1.86	NS	NS	0.023
Heat rate beat/min	17 ± 15 (62)	18 ± 15 (66)	18 ± 12 (20)	0.05	NS	NS	0.001
Rate pressure product, mmHg/min	4,337 ± 3,024 (62)	4,600 ± 2,817 (66)	4,551 ± 2,573 (20)	0.23	NS	NS	0.003
Body temperature, °C	0.2 ± 0.5 (62)	0.3 ± 0.5 (66)	0.3 ± 0.5 (20)	0.72	NS	NS	0.010
Adjective Mood Rating Scale rating ΔE_max_							
Activity	3.2 ± 4.9 (62)	2 ± 5.7 (66)	1.4 ± 2.9 (20)	1.23	NS	NS	0.017
High mood	3.1 ± 3.3 (62)	2.7 ± 3.3 (66)	2.4 ± 2.1 (20)	0.42	NS	NS	0.006
Fear/depression	1.0 ± 3.6 (62)	1.4 ± 3.3 (66)	0.6 ± 2 (20)	0.42	NS	NS	0.006
DAT1 rs460000	GG	GT	TT	F	p value	p value^a^	η^2^
N	81	58	10				
Female, N [%]	43 [53]	32 [55]	1 [10]				
Drug experience, N [%]	31 [38]	17 [29]	7 [70]				
MDMA plasma concentration Cmax, ng/ml	230 ± 49 (81)	230 ± 49 (58)	199 ± 35 (10)	1.96	NS	NS	0.026
MDMA plasma concentration AUC_6_, ng*h/ml	967 ± 211 (81)	967 ± 196 (58)	851 ± 155 (10)	1.55	NS	NS	0.021
Visual Analog Scale rating ΔE_max_							
Any drug effect	72 ± 27 (81)	73 ± 27 (58)	74 ± 23 (10)	0.60	NS	NS	0.007
Good drug effect	71 ± 29 (81)	75 ± 28 (58)	75 ± 27 (10)	0.59	NS	NS	0.007
Bad drug effect	17 ± 22 (81)	16 ± 26 (58)	13 ± 36 (10)	0.03	NS	NS	0.000
Drug liking	71 ± 31 (81)	77 ± 27 (58)	78 ± 21 (10)	0.95	NS	NS	0.012
Stimulated	64 ± 34 (81)	62 ± 36 (58)	51 ± 30 (10)	0.29	NS	NS	0.004
High mood	69 ± 32 (81)	68 ± 35 (58)	73 ± 29 (10)	0.40	NS	NS	0.005
Concentration	9.0 ± 15 (81)	7.4 ± 16 (58)	11 ± 20 (10)	0.29	NS	NS	0.004
Talkative	23 ± 19 (81)	19 ± 19 (58)	20 ± 11 (10)	0.55	NS	NS	0.007
Appetite	-4.8 ± 28 (36)	-6.4 ± 34 (29)	-24 ± 27 (7)	1.51	NS	NS	0.041
Tired	24 ± 33 (54)	16 ± 31 (46)	12 ± 29 (9)	1.00	NS	NS	0.018
Fear	8 ± 19 (55)	2.5 ± 5.8 (35)	13 ± 25 (7)	1.86	NS	NS	0.038
Happy	27 ± 18 (60)	28 ± 20 (41)	21 ± 14 (5)	0.05	NS	NS	0.001
Want to be hugged	10 ± 16 (45)	19 ± 21 (29)	0 ± 0 (3)	2.36	NS	NS	0.056
Want to hug	11 ± 16 (45)	19 ± 20 (29)	0 ± 0 (3)	2.26	NS	NS	0.053
Vital signs parameters ΔE_max_							
Systolic blood pressure, mmHg	23 ± 14 (81)	25 ± 11 (58)	24 ± 12 (10)	0.49	NS	NS	0.006
Diastolic blood pressure, mmHg	13 ± 9 (81)	14 ± 8 (58)	18 ± 17 (10)	2.97	0.05	NS	0.037
Mean arterial pressure, mmHg	16 ± 10 (81)	18 ± 8 (58)	22 ± 13 (10)	2.90	0.06	NS	0.036
Heat rate beat/min	16 ± 15 (81)	19 ± 15 (58)	16 ± 7 (10)	0.66	NS	NS	0.009
Rate pressure product, mmHg/min	4,219 ± 2,944 (81)	4,902 ± 2,877 (58)	3,963 ± 1,624 (10)	1.03	NS	NS	0.013
Body temperature, °C	0.2 ± 0.5 (81)	0.3 ± 0.4 (58)	0.3 ± 0.6 (10)	0.73	NS	NS	0.010
Adjective Mood Rating Scale rating ΔE_max_							
Activity	2.5 ± 5.2 (81)	2.4 ± 5.3 (58)	2.0 ± 2.7 (10)	0.02	NS	NS	0.000
High mood	2.8 ± 3.3 (81)	3.0 ± 3.2 (58)	2.2 ± 1.3 (10)	0.26	NS	NS	0.004
Fear/depression	0.9 ± 3 (81)	1.4 ± 3.7 (58)	1.2 ± 1.5 (10)	0.46	NS	NS	0.006
DAT1 rs463379	CC	CG	GG	F	p value	p value^a^	η^2^
N	94	48	7				
Female, N [%]	52 [55]	22 [46]	2 [29]				
Drug experience, N [%]	33 [35]	20 [42]	2 [29]				
MDMA plasma concentration Cmax, ng/ml	232 ± 50 (94)	221 ± 47 (48)	221 ± 37 (7)	0.98	NS	NS	0.013
MDMA plasma concentration AUC_6_, ng*h/ml	971 ± 200 (94)	937 ± 214 (48)	958 ± 185 (7)	0.46	NS	NS	0.006
Visual Analog Scale rating ΔE_max_							
Any drug effect	73 ± 26 (94)	71 ± 28 (48)	72 ± 20 (7)	0.01	NS	NS	0.000
Good drug effect	75 ± 28 (94)	69 ± 31 (48)	67 ± 26 (7)	0.68	NS	NS	0.009
Bad drug effect	18 ± 26 (94)	14 ± 21 (48)	9.1 ± 17 (7)	0.57	NS	NS	0.007
Drug liking	77 ± 27 (94)	69 ± 33 (48)	65 ± 27 (7)	1.27	NS	NS	0.016
Stimulated	61 ± 35 (94)	66 ± 33 (48)	57 ± 37 (7)	0.71	NS	NS	0.009
High mood	70 ± 33 (94)	67 ± 32 (48)	64 ± 33 (7)	0.14	NS	NS	0.002
Concentration	9.1 ± 17 (94)	7.9 ± 15 (48)	5.4 ± 11 (7)	0.23	NS	NS	0.003
Talkative	23 ± 19 (94)	18 ± 18 (48)	17 ± 20 (7)	0.86	NS	NS	0.011
Appetite	-6.8 ± 33 (53)	-6.7 ± 26 (15)	-15 ± 16 (4)	0.11	NS	NS	0.003
Tired	19 ± 32 (72)	22 ± 34 (32)	19 ± 23 (5)	0.23	NS	NS	0.004
Fear	6.4 ± 18 (66)	5 ± 9.5 (27)	14 ± 10 (4)	0.56	NS	NS	0.012
Happy	28 ± 19 (65)	25 ± 19 (38)	24 ± 25 (3)	0.32	NS	NS	0.006
Want to be hugged	16 ± 20 (41)	8.6 ± 15 (33)	20 ± 26 (3)	1.85	NS	NS	0.044
Want to hug	16 ± 19 (41)	8.8 ± 14 (33)	22 ± 26 (3)	2.12	NS	NS	0.050
Vital signs parameters ΔE_max_							
Systolic blood pressure, mmHg	23 ± 13 (94)	24 ± 13 (48)	26 ± 15 (7)	0.35	NS	NS	0.005
Diastolic blood pressure, mmHg	14 ± 10 (94)	13 ± 9 (48)	12 ± 6 (7)	0.16	NS	NS	0.002
Mean arterial pressure, mmHg	17 ± 10 (94)	17 ± 9 (48)	16 ± 7 (7)	0.11	NS	NS	0.001
Heat rate beat/min	17 ± 14 (94)	17 ± 13 (48)	22 ± 21 (7)	0.32	NS	NS	0.004
Rate pressure product, mmHg/min	4,500 ± 2,891 (94)	4,239 ± 2,512 (48)	5,607 ± 4,506 (7)	0.66	NS	NS	0.009
Body temperature, °C	0.2 ± 0.5 (94)	0.3 ± 0.5 (48)	0.2 ± 0.6 (7)	0.84	NS	NS	0.011
Adjective Mood Rating Scale rating ΔE_max_							
Activity	2.4 ± 5.7 (94)	2.1 ± 4.0 (48)	4.0 ± 2.4 (7)	0.42	NS	NS	0.006
High mood	3 ± 3.1 (94)	2.5 ± 3.3 (48)	3.7 ± 3.5 (7)	0.63	NS	NS	0.009
Fear/depression	1.2 ± 3.2 (94)	1.4 ± 3.6 (48)	-1.4 ± 3.6 (7)	2.25	NS	NS	0.030
N	7	47	93				
Female, N [%]	2 [29]	21 [45]	51 [55]				
Drug experience, N [%]	2 [29]	20 [43]	32 [34]				
MDMA plasma concentration Cmax, ng/ml	221 ± 37 (7)	221 ± 47 (47)	232 ± 50 (93)	0.86	NS	NS	0.012
MDMA plasma concentration AUC_6_, ng*h/ml	958 ± 185 (7)	934 ± 215 (47)	970 ± 200 (93)	0.49	NS	NS	0.007
Visual Analog Scale rating ΔE_max_							
Any drug effect	72 ± 20 (7)	71 ± 28 (47)	73 ± 26 (93)	0.00	NS	NS	0.000
Good drug effect	67 ± 26 (7)	69 ± 31 (47)	75 ± 28 (93)	0.60	NS	NS	0.008
Bad drug effect	9.1 ± 17 (7)	13 ± 21 (47)	18 ± 27 (93)	0.62	NS	NS	0.008
Drug liking	65 ± 27 (7)	69 ± 33 (47)	77 ± 27 (93)	1.19	NS	NS	0.016
Stimulated	57 ± 37 (7)	65 ± 33 (47)	61 ± 35 (93)	0.68	NS	NS	0.009
High mood	64 ± 33 (7)	67 ± 32 (47)	70 ± 33 (93)	0.10	NS	NS	0.001
Concentration	5.4 ± 11 (7)	8.1 ± 15 (47)	9.2 ± 17 (93)	0.23	NS	NS	0.003
Talkative	17 ± 20 (7)	19 ± 18 (47)	23 ± 19 (93)	0.64	NS	NS	0.009
Appetite	-15 ± 16 (4)	-6.7 ± 26 (15)	-6.8 ± 33 (53)	0.11	NS	NS	0.003
Tired	19 ± 23 (5)	22 ± 34 (32)	19 ± 32 (72)	0.23	NS	NS	0.004
Fear	14 ± 10 (4)	5 ± 9.5 (27)	6.4 ± 18 (66)	0.56	NS	NS	0.012
Happy	24 ± 25 (3)	25 ± 19 (37)	28 ± 18 (64)	0.23	NS	NS	0.004
Want to be hugged	20 ± 26 (3)	7.7 ± 14 (32)	15 ± 19 (40)	2.02	NS	NS	0.050
Want to hug	22 ± 26 (3)	8.1 ± 14 (32)	15 ± 19 (40)	2.22	NS	NS	0.054
Vital signs parameters ΔE_max_							
Systolic blood pressure, mmHg	26 ± 15 (7)	24 ± 14 (47)	23 ± 13 (93)	0.27	NS	NS	0.004
Diastolic blood pressure, mmHg	12 ± 6 (7)	13 ± 9 (47)	14 ± 10 (93)	0.15	NS	NS	0.002
Mean arterial pressure, mmHg	16 ± 7 (7)	17 ± 9 (47)	17 ± 10 (93)	0.09	NS	NS	0.001
Heat rate beat/min	22 ± 21 (7)	17 ± 13 (47)	17 ± 14 (93)	0.30	NS	NS	0.004
Rate pressure product, mmHg/min	5,607 ± 4,506 (7)	4,245 ± 2,539 (47)	4,514 ± 2,903 (93)	0.64	NS	NS	0.009
Body temperature, °C	0.2 ± 0.6 (7)	0.3 ± 0.5 (47)	0.2 ± 0.5 (93)	0.67	NS	NS	0.009
Adjective Mood Rating Scale rating ΔE_max_							
Activity	4.0 ± 2.4 (7)	2.1 ± 4.1 (47)	2.4 ± 5.7 (93)	0.41	NS	NS	0.006
High mood	3.7 ± 3.5 (7)	2.5 ± 3.3 (47)	2.9 ± 3.1 (93)	0.57	NS	NS	0.008
Fear/depression	-1.4 ± 3.6 (7)	1.4 ± 3.6 (47)	1.2 ± 3.2 (93)	2.27	NS	NS	0.031

N, number of subjects; SD, standard deviation; NS, not significant; Δ, values are change scores from placebo; ^a^p value additionally corrected for multiple comparisons according to the Nyholt method; η^2^, eta square; *, uncorrected p < 0.05.

**Table 3:** Effects of the variable-number tandem repeat polymorphism in the dopamine receptor D4 gene on the maximal response to 125 mg MDMA (mean ± SD (N) and statistics) corrected with MDMA AUC_6_ (exclusive plasma concentrations).

DRD4 VNTR	≤8 Repeats	>8 Repeats	F	p value	p value^a^	η^2^
N	87	59				
Female, N [%]	44 [51]	31 [53]				
Drug experience, N [%]	31 [36]	22 [37]				
MDMA plasma concentration Cmax, ng/ml	229 ± 44 (87)	226 ± 55 (59)	0.16	NS	NS	0.001
MDMA plasma concentration AUC_6_, ng*h/ml	965 ± 189 (87)	948 ± 221 (59)	0.25	NS	NS	0.002
Visual Analog Scale rating ΔE_max_						
Any drug effect	74 ± 26 (87)	71 ± 26 (59)	0.35	NS	NS	0.002
Good drug effect	73 ± 30 (87)	73 ± 26 (59)	0.01	NS	NS	0.000
Bad drug effect	17 ± 23 (87)	15 ± 27 (59)	0.08	NS	NS	0.001
Drug liking	74 ± 31 (87)	75 ± 25 (59)	0.12	NS	NS	0.001
Stimulated	63 ± 35 (87)	63 ± 34 (59)	0.07	NS	NS	0.000
High mood	68 ± 34 (87)	71 ± 31 (59)	0.51	NS	NS	0.003
Concentration	8.2 ± 17 (87)	9.0 ± 15 (59)	0.09	NS	NS	0.001
Talkative	20 ± 19 (87)	23 ± 19 (59)	1.27	NS	NS	0.008
Appetite	-5.8 ± 33 (47)	-10 ± 26 (25)	0.41	NS	NS	0.006
Tired	24 ± 32 (68)	13 ± 32 (41)	2.63	NS	NS	0.023
Fear	6.6 ± 18 (56)	5.6 ± 14 (38)	0.08	NS	NS	0.001
Happy	26 ± 20 (59)	30 ± 17 (44)	1.33	NS	NS	0.012
Want to be hugged	13 ± 19 (40)	13 ± 18 (34)	0.01	NS	NS	0.000
Want to hug	14 ± 19 (40)	13 ± 17 (34)	0.02	NS	NS	0.000
Vital signs parameters ΔE_max_						
Systolic blood pressure, mmHg	25 ± 12 (87)	22 ± 13 (59)	1.24	NS	NS	0.008
Diastolic blood pressure, mmHg	14 ± 9 (87)	13 ± 10 (59)	0.11	NS	NS	0.001
Mean arterial pressure, mmHg	17 ± 9 (87)	17 ± 10 (59)	0.11	NS	NS	0.001
Heat rate beat/min	18 ± 15 (87)	17 ± 14 (59)	0.03	NS	NS	0.000
Rate pressure product, mmHg/min	4,561 ± 2,967 (87)	4,393 ± 2,746 (59)	0.06	NS	NS	0.000
Body temperature, °C	0.3 ± 0.5 (87)	0.2 ± 0.5 (59)	0.19	NS	NS	0.001
Adjective Mood Rating Scale rating ΔE_max_						
Activity	2.3 ± 5.2 (87)	2.7 ± 4.9 (59)	0.26	NS	NS	0.002
High mood	2.8 ± 3.3 (87)	3.0 ± 3.0 (59)	0.18	NS	NS	0.001
Fear/depression	1.1 ± 3.7 (87)	0.9 ± 3 (59)	0.10	NS	NS	0.001

N, number of subjects; SD, standard deviation; NS, not significant; Δ, values are change scores from placebo; ^a^p value additionally corrected for multiple comparisons according to the Nyholt method; η^2^, eta square.

The 60-item Likert-type scale of the short version of the Adjective Mood Rating Scale (AMRS) (59) was administered before and 1.25, 2, and 5 h after MDMA or placebo administration. The AMRS contains subscales for activity, well-being, and anxiety–depression.

### Genotyping

Genomic DNA was extracted from whole blood using the QIAamp DNA Blood Mini Kit (Qiagen, Hombrechtikon, Switzerland) and automated QIAcube system. SNP genotyping was performed using commercial TaqMan SNP genotyping assays (LuBio Science, Lucerne, Switzerland). We assayed the following SNPs: DRD2/ankyrin repeat and kinase domain containing 1 (ANKK1) SNPs rs1800497 (assay: C___7486676_10), DRD2 rs6277 (assay: C__11339240_10), and rs1079597 (assay: C___2278884_10), and DAT1 SNPs rs6347 (assay: C___8769902_10) and rs11133767 (assay: C___3024834_10) and rs11564774 (assay: C__25761679_10) and rs460000 (assay: C___3284837_10) and rs463379 (assay: C___3284827_10). We also used the following method to genotype the polymorphisms in DRD4 exon III VNTR, DAT1 3’UTR VNTR rs28363170, and DAT1 Intron 8 (5/6) VNTR rs3836790. Genotypes were determined by polymerase chain reaction (PCR) using 2.5, 1.25, and 1.25 units of HotStarTaq DNA polymerase (QIAGEN Instruments AG, Hombrechtikon, Switzerland), respectively; 1.5 µl PCR Buffer 10x each (15 mM Mg^2+^; QIAGEN Instruments AG, Hombrechtikon, Switzerland); 0.25, 1, and 1 µl dNTP Mix (40 mM), respectively; and primer set *5’-GCGACTACGTGGTCTACTCG* and *5’-AGGACCCTCATGGCCTTG, 5’-TGTGGTGTAGGGAACGGCCTGAG* and *5’-CTTCCTGGAGGTCACGGCTCAAGG*, and *5’-GCATGTGGATGTGTTCTTGCA* and *5’-TCATCCCAGGGACATCTGCTA* (both 1 µl, both 0.5 µl, and both 0.5 µl, respectively) in a total reaction volume of 15 µl each. The following temperature profile was applied in a T100 thermal cycler (Bio-Rad, Cressier, Switzerland): for DRD4 (Exon III VNTR): initial activation step of 95°C (15 min) and 30 cycles of 98°C (60 s), 67.5°C (60 s), and 72°C (60 s), with final extension at 72°C (5 min); for DAT1 (3’UTR VNTR) rs28363170 and (intron8 5/6 VNTR) rs3836790: initial activation step of 95°C (15 min) and 30 cycles of 98°C (25 s), 95°C (35 s), and 72°C (45 s), with final extension at 72°C (5 min). The sizes of the resulting PCR products were assessed by 3.5% (for DRD4 exon III VNTR) and 2.5% (for DAT1 3’UTR VNTR rs28363170 and Intron 8 VNTR rs3836790) agarose gel electrophoresis. Amplicons of the DRD4 (Exon III VNTR in chromosome 11) of 379 bp were designated as 2 repeats (2R), and amplicons of every additional 48 bp were designated as 2+x times 48 bp variants [up to 9R (with 379 bp + 7 × 48 bp = 715 bp)]. Four and 7-repeat amplicons were the most common forms. Complete genotype and allele distributions are depicted in **Supplementary Table S1**. For the analysis, groups were made with cumulative ≤8 repeats or cumulative >8 repeats in both alleles. Amplicons of the DAT1 (3’UTR VNTR) rs28363170 of 448 bp were designated as 9 repeats (9R), and amplicons of 488 bp were designated as 10R. Individuals possessing other repeats were excluded from the analysis. Amplicons of the DAT1 (intron 8 5/6 VNTR) rs3836790 of 295 bp were designated as 5 repeats (5R), and amplicons of 325 bp were designated as 6 repeats (6R). The pairwise linkage disequilibrium (LD) and relative physical location of the determined SNPs on chromosome 11 (DRD2) and 5 (DAT1) are shown in **Supplementary Figure 1**. The tested genetic variants were consistent with Hardy–Weinberg equilibrium (*p* > 0.05).

### Statistical Analysis

The statistical analyses were performed using Statistica 12 software (StatSoft, Tulsa, OK, USA). For repeatedly measured data, peak effects (E_max_) and areas under the effect-time curve (AUEC) from 0- to 6-h values were determined for MDMA and placebo. Differences in E_max_ (Δ; MDMA-placebo) were then analyzed using one-way analysis of variance (ANOVA), with genotype as the between-group factor. The level of significance was set at *p* < 0.05. The Nyholt correction method was used to account for multiple comparisons and displayed separately in all tables (60). We thereby corrected for 17 subjective effect ratings (VAS+AMRS), and six vital parameters. In addition, this was then corrected for each of the 11 polymorphisms tested, resulting in (17 + 6) × 11 = 253 variables and an effective number of independent variables (V_eff_) of 183.6 according to Nyholt. Consequently, this led to a corrected significance threshold of p < 0.00027 to keep Type I error rate at 5%. To account for differences in plasma concentrations of MDMA that were caused by differences in body weight, dosing, or metabolizing enzymes (17, 18), the area under the MDMA plasma concentration–time curve from 0 to 6 h (AUC) was included as a covariate in the ANOVAs, and we report the corrected statistics. Additionally, modulatory effects of sex were explored by adding sex as a between-subjects factor in the ANOVAs (sex × genotype). E_max_ values were obtained directly from the observed data. AUC and AUEC values were calculated using the linear-log trapezoidal method in Phoenix WinNonlin 6.4 (Certara, Princeton, NJ, USA). The primary analysis was performed using an additive genotype model approach for SNPs. Recessive or dominant model analysis was also performed, the results of which are reported only when the additive model was initially significant.

## Results

MDMA significantly altered all tested VAS and AMRS E_max_ values. Subjects did not significantly differ in MDMA plasma concentration or previous drug experience across genotype groups, with the exception of DAT1 rs11133767. Participants carrying two T-alleles showed disproportionately more illicit drug experiences than carriers of the C-allele (70% vs. 31%, respectively; χ^2^ = 11.2, p < 0.001).

The influence of polymorphisms within genes coding for the DRD2, DAT1, and DRD4 on the maximal acute subjective and autonomic effects of MDMA is shown in **Tables 1**–**3**, respectively. **Supplementary Table S2** shows the data for the total response to MDMA over time (AUEC). **Supplementary Tables S3** and **S4** show the uncorrected statistics for E_max_ and AUEC, respectively. Homozygous A-allele carriers of the DRD2 rs1800497 showed a higher score in VASs “talkative” (F_1,147_ = 4.23, p < 0.05) and in AMRSs “activity” and “high mood” (F_1,147_ = 4.62, p < 0.05 and F_1,147_ = 4.50, p < 0.05, respectively) compared to carriers of the G-allele. Subjects with two 9R-alleles of the DAT1 rs28363170 had a higher MDMA-induced increase in diastolic blood pressure and MAP compared to subjects with a 10R-allele (F_1,141_ = 7.12, p < 0.01 and F_1,141_ = 6.56, p < 0.05, respectively). Regarding the DAT1 rs3836790, MDMA produced a higher increase in MAP in individuals homozygous for the 5R-allele compared to 6R-allele carriers (F_1,144_ = 4.31, p < 0.05).

Nyholt correction for multiple comparisons yielded statistics indicating that the genetic polymorphisms had no significant effect on the subjective and autonomic parameters. Sex did not significantly modulate the results.

## Discussion

The current study expands previous research on whether the acute effects of MDMA are modulated by common genetic polymorphisms in pharmacological targets of MDMA. So far, the focus lied on the role of the NE and 5-HT system genetics in the acute effects of MDMA (22, 23). This is the first study to concentrate on a selection of genetic polymorphisms within the human DA system (namely, D_2_, D_4_, and DAT).

Action on the DA system is thought to be crucial for the effects of most psychostimulant substances (6, 24, 61), and pharmacogenetic studies demonstrated that different phenotypes are affected by various DA genotypes. As for MDMA, however, none of the herein investigated genetic polymorphisms significantly altered the acute effects after consideration of Type I error correction.

Nevertheless, this missing link between DA genetic variations and MDMA-related phenotypes might not solely be caused by a lack of genetic influence on the MDMA effects but rather the potentially minor role of DA in MDMA effects. Although MDMA is an amphetamine, it acts mainly on the 5-HT system and therefore leads to its classification as an entactogen (7, 62).

The present study has limitations. Although this analysis was done using the largest sample of healthy human subjects who received MDMA in placebo-controlled studies, the sample size is still relatively small when considering the partially small rare allele groups and mostly weak effect sizes for the influence of genetic variants on the MDMA response. This is especially influencing spurious, uncorrected effects (i.e., the AA carrier group for the SNP DRD2/ANKK1 rs1800497 with N = 2). Larger cohorts might show a more balanced sample distribution, which might lead to different results. Additionally, the study was conducted in healthy volunteers with a single dose of 125 mg MDMA. Therefore, the findings may not be applied to other populations and situations, such as psychiatric patients and the use of higher doses of MDMA. Furthermore, SNPs in genes of other targets of MDMA may also be involved. However, we corrected for the modulatory effects of known genetic variants that influence the metabolism of MDMA (17, 18) by taking interindividual differences in plasma MDMA concentrations into account. We also might have missed some relevant genetic polymorphisms. A novel potentially functional SNP within the DAT1 has been described in recent research. However, the SNP showed no significant alteration in the inhibition of DA uptake by MDMA in human embryonic kidney 293 cells (63). We have also not tested for rare haplotypes because a haplotype approach may lead to very small groups and more potential statistical artifacts. However, a haplotype suggested by Brewer et al., which consists of rs28363170 10/10 genotype and at least one rs3836790 5R-allele carriers, showed a reduced subjective response to cocaine compared to others (40). The same haplotype showed no effect in the present study. In fact, uncorrected results even implied opposite and incoherent effects, with 10R carriers showing lower MDMA-induced MAP changes and 5/5 carriers showing higher MAP changes than subjects with the 9/9 genotype or a 6R-allele, respectively. This incoherency may be attributable to the different substances used (cocaine vs. MDMA) and different cohorts (80% males of African descent vs. the sex-balanced sample of European descent) (40). Additionally, MDMA may interact with a different binding site on the DAT compared to other stimulants like cocaine (64). Finally, previous drug experiences were not equally distributed among DAT1 rs11133767 genotype groups, and effects might slightly depend on previous substance use experiences. Because of the involvement of DA in addiction, subjects carrying a TT genotype may be more prone to illicit substance use (65). Apart from this finding, given that our cohort included mostly drug-naive subjects with limited drug use experience, some alleles associated with increased drug use might even be underrepresented. However, the tested variants were consistent with the Hardy–Weinberg equilibrium and comparable with frequencies found in European genome databases.

We conclude that the present findings align with previous studies in that variations in genes coding for players of the monoaminergic systems are unlikely to explain interindividual variations in the acute effects of MDMA in humans.

## Data Availability Statement

The datasets for this manuscript are not publicly available because the individual genotyping consent did not include storing in public repository. Requests to access the datasets should be directed to Matthias Liechti, Matthias.liechti@usb.ch. 

## Ethics Statement

The studies involving human participants were reviewed and approved by Ethikkommission Nordwest- und Zentralschweiz (EKNZ). The patients/participants provided their written informed consent to participate in this study.

## Author Contributions

PV analyzed the data and wrote the manuscript. ML conceived the study, obtained funding, and wrote the manuscript.

## Funding

This study was supported by the Swiss National Science Foundation (grant no. 320030_170249).

## Conflict of Interest

The authors declare that the research was conducted in the absence of any commercial or financial relationships that could be construed as a potential conflict of interest.
