# Supplementary material for: No Influence of Dopamine System Gene Variations on Acute Effects of MDMA
Source: Front Psychiatry. 2019 Oct 24;10:755. doi: 10.3389/fpsyt.2019.00755 (PMC6821788; doi:10.3389/fpsyt.2019.00755)
Supplement: Supplementary file 5 [file Image_1.pdf]

Supplementary Material

Supplementary Figures

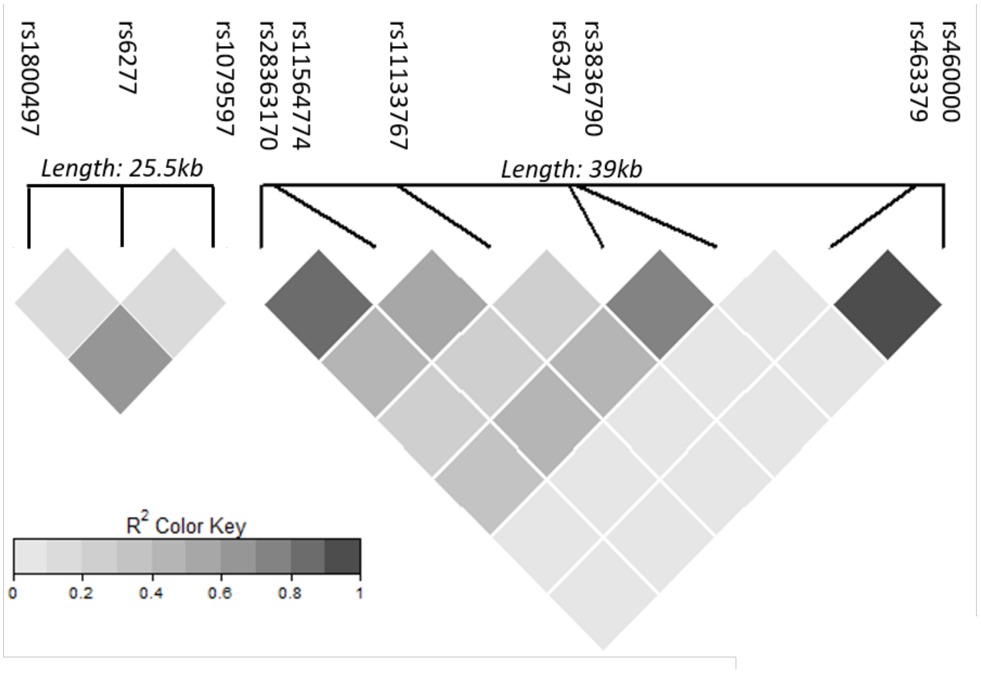

Supplementary Figure 1. The pairwise linkage disequilibrium (LD) and relative physical location of the determined SNPs of DRD2 on chromosome 11 (left) and of DAT1 on chromosome 5 (right). Graphics and analysis are performed according to Shin et al. (Shin et al., 2006).

Shin, J.-H., Blay, S., McNeney, B., and Graham, J. (2006). LDheatmap: An R Function for Graphical Display of Pairwise Linkage Disequilibria Between Single Nucleotide Polymorphisms. *2006* 16(Code Snippet 3)**,** 9. doi: 10.18637/jss.v016.c03.
